# Surfactant Protein A in particles in exhaled air (PExA), bronchial lavage and bronchial wash - a methodological comparison

**DOI:** 10.1186/s12931-019-1172-1

**Published:** 2019-09-26

**Authors:** Annelie F. Behndig, Ekaterina Mirgorodskaya, Anders Blomberg, Anna-Carin Olin

**Affiliations:** 1 Department of Public Health and Clinical Medicine, Division of Medicine, Umeå University, Umeå, Sweden; 2 Proteomics Core Facility, Sahlgrenska Academy, University of Gothenburg, Gothenburg, Sweden; 3 Occupational and Environmental Medicine, Inst of Medicine, Sahlgrenska Academy, University of Gothenburg, Box 414, 405 30, Gothenburg, Sweden

## Abstract

**Introduction:**

At present, there are few methods available for monitoring respiratory diseases affecting distal airways. Bronchoscopy is the golden standard for sampling the lower airways. The recently developed method for collecting non-volatile material from exhaled air – PExA (Particles in Exhaled air) is a promising new tool, but no direct comparison between the two methods has yet been performed. The aim of the present study was to compare sampling using PExA with bronchial wash (BW) representing the larger more proximal airways and broncho-alveolar lavage (BAL) representing the distal airways.

**Methods:**

15 healthy non-smoking subjects (7 female/8 male), age 28 ± 4 years, with normal lung function were included in the study. PExA-sampling (2 × 250 ng particles) and bronchoscopy with BW (2 × 20 ml) and BAL (3 × 60 ml sterile saline) was performed. Albumin and Surfactant Protein A (SP-A) were analyzed with ELISA, and analyses of correlation were performed.

**Results:**

A significant association was found between BAL-fluid albumin and PExA-albumin (r_s_:0.65 *p* = 0.01). There was also an association between SP-A in PExA and BAL, when corrected for albumin concentration (r_s_:0.61, *p* = 0.015). When correlating concentrations of albumin and SP-A in bronchial wash and PExA respectively, no associations were found.

**Conclusions:**

This is the first direct comparison between the bronchoscopy-based BW/BAL-fluids and material collected using the PExA methodology. Both albumin and albumin-corrected SP-A concentrations were significantly associated between BAL and PExA, however, no such association was found in either marker between BW and PExA. These results indicate that the PExA method samples the distal airways. PExA is thus considered a new promising non-invasive assessment for monitoring of the distal airways.

## Background

There is a great need for new non-invasive methods to monitor pathology in small airways. Already in 1970, the small airways were denoted “the quiet zone” of the lung by Mead [1]. Since then, new tools have been developed to measure structural changes, such as impulse oscillometry (IOS), and there has been an incredible development in imaging. At the same time, sampling the airways still remains difficult, and is currently limited to bronchoscopy or sputum induction. Bronchoscopy has a clear advantage, given the possibility to inspect the airways and to take samples directly from a suspected lesion. However, the invasiveness of bronchoscopy limits the possibilities of repeated sampling within a short period. Moreover, easy-to-perform methods for screening of early airway pathological processes are missing, especially those offering sample collection to study airway inflammation or abnormalities.

During the last decades, analysis of exhaled air has gained increasing interest and, for assessing small airways, modeling of alveolar nitric oxide (NO) levels has been used in a number of studies [2]. The modeling of alveolar NO is still an issue, and recent data showed that the calculated levels are influenced by structural changes of the small airways and the degree of diffusion of air in peripheral direction, making interpretation complex [3]. Detecting other volatiles in breath is also emerging as an interesting research field, where an unbiased approach based on pattern recognition and artificial intelligence (AI) is of great interest [4]. However, this method reflects both local and systemic effects, and does not specifically allow a characterization of pathological processes in small airways.

To meet this gap, a new method to sample airway lining fluid from the small airways has been developed [5]. The method is based on sampling of small droplets/particles in the exhaled aerosol. When small airways re-open after a deep exhalation, small liquid droplets carrying the airway lining fluid are formed and those can be sampled in exhaled breath by a new instrument, the PExA®, specially developed for this purpose [6]. The instrument collects droplets in the size-range of 0.5–4.2 μm, thereby avoiding sampling of larger droplets, formed in the more central and upper airways during exhalation. Calculation of the number of sampled particles at different size-fractions allows one to estimate the volume of sampled airway lining fluid and makes quantification of measured components possible. A low intra-individual variation of measured analytes in PExA samples has encouraged further exploration [7].

The PExA sample (hereafter called PEx) mainly consists of lipids, in particular phospholipids [8] from the surfactant, but around 20% are proteins [9]. The major proteins in PEx are immunoglobulins, followed by albumin, that constitutes around 25% of the proteins in the sample [9].

A few clinical studies have been carried out using the PExA method, mainly focusing on the evaluation of Surfactant Protein A (SP-A). SP-A is the most abundant lung-specific protein and plays a vital role in host defense [10], as it binds to inhaled pathogens and particles that are too small to directly be recognized by alveolar macrophages and dendritic cells. Hence, it seems as an interesting biomarker to explore further, especially as it easily can be measured in breath, a highly relevant matrix for biomarkers for lung-disease. In COPD [11], broncho-obliterans syndrome (BOS) in lung-transplanted patients [12] and gastro-esophageal reflux [13], SP-A concentrations in PEx were lower than in healthy subjects, whereas they were not altered in mild-moderate asthma [14]. On the other hand, in asthma with small airway involvement, SP-A concentrations were lower, suggesting its importance to identify a specific sub-phenotype with more severe disease and lower quality of life [15].

In the present study, we aimed to assess how the levels of SP-A in samples collected by PExA method resemble that of bronchial wash and broncho-alveolar lavage, representing larger proximal airways and distal airways, respectively. The composition of the material collected using PExA has not been previously compared with that of BW/BAL fluid, and such comparison is an important step in the validation of the method. Our hypothesis was that the levels of SP-A in PEx would be more strongly associated with those in BAL compared to BW, given the more distal origin of BAL fluid. In addition to SP-A, we measured albumin in all samples, to compare how the two analytes behaved in the different matrices.

## Methods

### Subjects and study design

15 healthy nonsmoking subjects (7 female/8 male), age 28 ± 4 years with normal lung function and normal BMI (mean 23.6 ± 2.) were included in the study. All individuals performed PExA-sampling and blood sampling on day one and bronchoscopy the following day.

All participants gave their written informed consent and the study was approved by the Ethical Committee at Umeå University in Sweden.

### Bronchoscopy

Bronchoscopy was performed at the Department of Medicine, Division of Respiratory Medicine and Allergy, University Hospital, Umeå, Sweden. Premedication with 1.0 mg of atropine was given subcutaneously 30 min before the procedure. Topical anaesthesia was achieved using lidocaine. A flexible video bronchoscope (Olympus BF 1 T160, Tokyo, Japan) was inserted through the mouth via a mouthpiece with the subjects in the supine position. Airway lavages were performed in the lingual lobe of the left lung or the right middle lobe. To achieve bronchial wash, 2 × 20 ml of sterile sodium chloride (0.9%), pH 7.3 at 37 °C was infused and gently sucked back after each infusion and pooled into the same tube placed on ice. Broncho-alveolar lavage (BAL) was performed by infusing three aliquots of 60 ml of sterile sodium chloride, sucked back after each infusion and pooled into a tube placed in iced water. All lavage samples were filtered through a nylon filter (pore diameter 100 μm) and centrifuged at 400 g for 15 min. Cell pellets were re-suspended in phosphate-buffered saline at a cell concentration of 10^6^ cells/ml. Differential cell counts were performed on cyto-centrifuge preparations stained with May-Grünwald Giemsa and 400 cells per slide were counted.

### Sampling of PExA

The method to collect particles in exhaled air has been described earlier [7], using an instrument developed specifically for this purpose (PExA™, Sahlgrenska University Hospital, Gothenburg, Sweden). Before sample collection starts, participants breathed filtered air for three minutes tidally to avoid contamination of ambient particles. A specific breathing maneuver, allowing for airway closure and re-opening to augment the number of exhaled particles, was applied. In this maneuver, the participants exhaled to residual volume, held their breath for three seconds, then inhaled sharply up to full inspiration, and finally exhaled to almost full expiration. Particles were only collected from the last exhalation of this maneuver. In between these breathing maneuvers, the participants breathed filtered air tidally for 30–60 s. In the present study, this breathing maneuver was repeated until 250 ng of PEx had been collected.

The total mass of the collected particles was calculated based on the number and size of the particles, assuming them to be spherical and have a density of 1000 kg/m^3^. [7] The exhaled particles were collected by impaction on a teflon filter (LCR Membrane Filter, Merck Millipore, Germany), which was divided into two halves immediately after sampling, and each half was stored in a polypropylene test tube (Screw cap micro tube, Sarstedt, Germany). The filters were immediately frozen at − 20 °C, and within four hours moved to − 80 °C, until analyses were performed.

### Immuno-detection of surfactant protein a (SP-A) and albumin

PEx, BAL, and BW samples were analyzed for every study participant. All collected sample were stored at − 80 °C and were prepared for immunoassay using the same extraction solvents and procedures.

#### Sample extraction

All samples were extracted using *Extraction Buffer*, a phosphate-buffered saline (PBS) buffer prepared as 10 mM Na Phosphate, 0.15 M NaCl, containing 1% bovine serum albumin, w/v and 0.05% Tween-20, v/v. Sample preparation procedures were designed to be similar for all samples (PEx and lavage samples), but were adopted according to the sample type.

For PEx samples, 140 μl *Extraction Buffer* was added to each sample Vials were spun down to ensure that sampling membranes were fully covered by the extraction solvent prior to incubation in a thermomixer. For BAL and BW samples, 10 μl of each sample was mixed with 990 μl of *Extraction Buffer* prior to incubation in a thermomixer.

After the addition of *Extraction Buffer*, samples were shaken at 400 rpm for 60 min at 37 °C in a thermomixer (Thermomixer comfort, Eppendorf; Eppendorf AG, Hamburg, Germany).

The extracted samples were stored at -20C prior to immunoassay and were analyzed within a week after extraction.

#### Immunoassay

SP-A and albumin quantifications were carried out using commercially available ELISA kits, human SP-A ELISA kit (RD191139200R, BioVendor, Czech Republic) and human albumin ELISA kit (E-80AL, Immunology Consultants Laboratory, Inc., USA), respectively. The assays were performed according to the manufacturer’s instructions, with minor modifications to the buffer composition and incubation times.

Extracted samples were further diluted directly prior to the immunoassays. Different dilution factors were used depending on the assay and sample type. After the final dilution step, all samples were in the same *Assay Buffer* composed of *Extraction Buffer* and the corresponding *ELISA sample diluent*, provided with each kit, in the ratio 1:2, v/v.

For SP-A assay, extracted samples were further diluted 3 times for PEx and 9 times for lavage samples.

For albumin ELISA, extracted PEx samples were diluted 3 times and extracted lavage samples were diluted 45 times.

All calibrants and controls were prepared and assayed in the same *Assay Buffer* as the study samples, i.e., 1:2, v/v mix of *Extraction Buffer* and the corresponding *ELISA sample diluent*. The plate incubation times were extended to 3 h for SP-A assay and to 1.5 h for albumin assay. The absorbance was read at 450 nm by a plate-reader from BioTek ELx-808UI (Highland Park, MI, USA) with 630 nm as a reference wavelength. The analyte concentrations were calculated automatically from the generated standard curves using the four-parameter algorithm. The limit of quantifications (LOQ) for the modified protocols, as determined by precision profile at 15% coefficient of variation (CV), were 0.5 ng/mL for the SP-A assay and 0.9 ng/mL for the albumin assay.

Quality control (QC) samples, prepared in duplicates, were used to control individual assays across the used quantification range. Extracted PEx samples were analyzed as singles. Lavage and blood samples were assayed in duplicates. For the SP-A ELISA, the average CVs for duplicates were 5.4% for BAL and 4.9% for BW samples. For the albumin ELISA, the average CVs were 5.0% for BAL, 6.6% for BW samples.

### Statistical analysis

Comparisons between two measurements were tested using the Wilcoxon signed rank test. Correlation analyses were performed using the Spearman’s rank order correlation. A *p*-value of < 0.05 was considered significant. Statistical analyses were performed using SPSS, version 24.0 (SPSS Inc., Chicago, IL).

## Results

Bronchoscopy and PExA sampling were achieved from all 15 individuals. BW recovery was 42 (40–46) % and BAL recovery 69 (61–74) %. The collected total mass of PEx was 255 (251–256) ng. Albumin and SP-A were quantified in PEx, BAL and BW fluids (Table 1). For PEx, the calculated protein amounts were related to total PEx mass and expressed as weight percent. The concentration of albumin and SP-A in BW and BAL-fluid did not differ between the two compartments (*p* = 0.82 and *p* = 0.28 respectively).

**Table 1:** Albumin and surfactant protein A in PEx-samples and bronchial lavages in healthy individuals

	PEx ng	PEx Wt%	BW µg/ml	BAL µg/ml
Albumin	16 (13–24)	6.3 (5.3–9.4)	25 (15–37)	29 (17–45)
SP-A	10 (8–11)	3.9 (3.1–4.3)	13 (9–16)	12 (10–21)

Collected PEx mass 255 (251–256) ng. *Wt%* weight percent, fraction of protein in relation to total PEx mass collected. Data presented as median with (IQR)

**Fig. 1:**
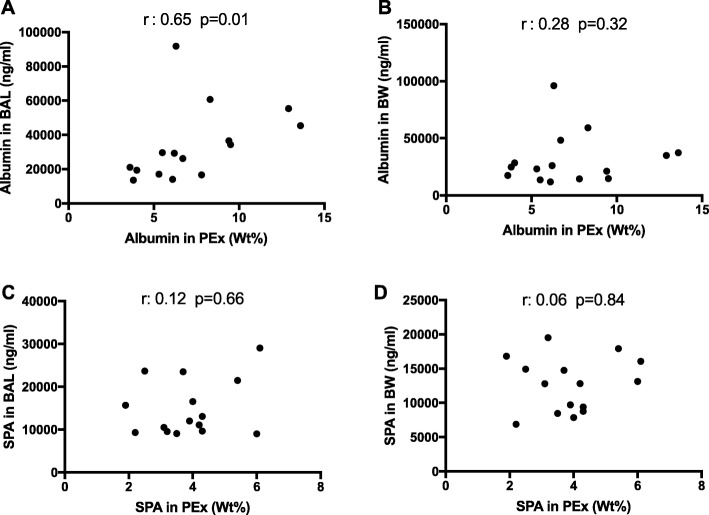
Associations between albumin and surfactant protein A concentrations measured in PExA and bronchoscopy samples from healthy individuals. **a** There was a significant association between albumin in BAL-fluid and PEx. **b**-**d** There was no significant association between SP-A in PEx and BAL or between albumin or SP-A between PEx and BW and BAL respectively. Spearman’s correlation. A *p*-value <0.05 was considered significant

There was a significant association between BAL-fluid albumin and PExA-albumin (r: 0.65 *p* = 0.01), (Fig. 1a) but not between BW-fluid albumin and PExA-albumin (Fig. 1b). No significant association was detected between SP-A in PEx, (expressed as ng or weight-percent) and BAL or BW, respectively (Fig. 1c-d). There was a significant association between BW-albumin and BAL-fluid albumin (r: 0.58 *p* = 0.02), but not between BW-SP-A and BAL-fluid-SP-A (r: 0.39 *p* = 0.15). When SP-A concentrations were corrected for albumin in each matrix, a strong association between SP-A in BW and in BAL-fluid was found (r: 0.87 *p* < 0.001).

As the concentration of SP-A in BW and BAL fluid may be influenced by dilution, a ratio between SP-A and albumin was calculated, both for BW, BAL and PEx (Table 2).

**Table 2:** Surfactant protein A in relation to albumin in samples collected with PExA and bronchoscopy (BW and BAL-fluid)

	PEx	BW	BAL
SP-A/Albumin	0.63 (0.37–0.79)	0.55 (0.24–0.82)	0.49 (0.33–0.80)

Fraction of SP-A in relation to albumin. Data presented as median with (IQR)

**Fig. 2:**
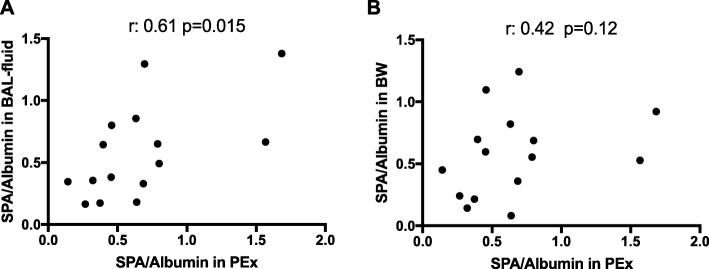
Associations between Surfactant Protein A collected with PExA and bronchoscopy (BW and BAL-fluid) after correction for albumin. **a** There was a significant association between SP-A in PEx and in BAL-fluid. **b** Again, no significant association was found between SP-A in PEx and in BW. Spearman’s correlation. A *p*-value <0.05 was considered significant

When SP-A concentrations were corrected for albumin in each matrix, there was a strong association between SP-A in PExA and in BAL-fluid (r: 0.61 *p* = 0.015) (Fig. 2a). Again, there was no association between the concentrations of albumin-corrected SP-A in bronchial wash and PExA (Fig. 2b).

## Discussion

For the first time, the protein-content in PEx has been compared with that of bronchoscopy-based bronchial wash and broncho-alveolar lavage, reflecting larger proximal and more distal small airways, respectively. We chose to compare the two most abundant proteins present in the airway fluids, albumin and SP-A. Although both proteins are abundant, they have different origins, i.e., album is the major serum protein produced in liver and SP-A is locally produced by alveolar type II cells. The major finding was that albumin-corrected SP-A levels were significantly associated between BAL and PEx, whereas no association was found between SP-A in BW and PEx. In accordance, there was an association between albumin concentrations in BAL and PEx, but not between BW and PEx**.**

As SP-A is mainly produced by type-II pneumocytes located in the very periphery of the airways, these findings indicate that the PEx sample origin is similar to the airways sampled with BAL, i.e., the distal airways. It is therefore likely that the SP-A concentration in the airway lining fluid is higher in the more distal airways compared to that of larger more proximal airways, but this has not previously been shown due to methodological difficulties. Whilst SP-A concentrations in BAL and PEx were significantly correlated (r_s_ = 0.61), there were still unexplained variation between the two matrices. This is likely due to the fact that the PEx sample is suggested to have a very distal origin, whereas BAL constitutes a mixed sample from both central and more distal airways. This is supported by the somewhat higher concentration ratios of SP-A/Albumin in PEx, (0.62 vs 0.49 in PEx and BAL respectively). The concentrations measured in BW were numerically higher than in BAL fluid, but were not statistically different. In this study only two proteins were measured and therefore some of the observation might be difficult to explain. To get a larger picture, global protein identification and quantification study aimed to compare these different airway sampling methods would have to be performed.

In the present study, SP-A concentrations in PEx and BAL correlated only when corrected for albumin levels. This is not surprising, as the protein levels in BAL fluid to a high extent are influenced by dilution. Whether or not correction for dilution in BAL fluid by using albumin should be performed is under debate, mainly as the albumin levels may be influenced by different airway/lung diseases. However, in the present study, in which only healthy participants were included, this bias is unlikely.

The detected levels of SP-A and albumin in PEx were in line with previous studies in healthy subjects [7, 11], as were BAL fluid levels, when ELISA-based methods were used for detection [16, 17].

A strength of the study is that it was performed by highly experienced bronchocopists, which limits variability in lavage recovery and enhances the comparability between the methods.

The study has some limitations; the number of subjects was limited, and a larger group would have been desirable, yet questionable from an ethical and practical point of view due to the invasiveness of the bronchoscopy procedure. The non-significant finding between SP-A concentrations in BW and BAL may be due to the limited number of subjects. But it seems anyhow likely that is less strong than that to BAL fluid, which was anticipated. It would have been of interest to study more biomarkers and their associations in all three matrices, but we were limited by resources.

## Conclusions

The present study shows a high agreement between SP-A concentrations in PEx samples and BAL fluid, when correcting for albumin levels, whereas SP-A levels in PEx and BW were not associated. This supports the hypothesis that PEx samples originate from the distal airways and that the protein composition of PEx, at least when it comes to these two markers, resembles that of BAL fluid. In comparison to airway lavage, PEx has the advantage of being a sample of undiluted airway lining fluid, where there is no need for correction. Its current limitation lies in the very low quantity of sample, and that there are no cells present in the sample. Nevertheless, PExA is considered a novel, non-invasive and easy-to-perform method to sample distal airway lining fluid. It thus has a potential to fill an important gap in the understanding of small airway pathology.

## Data Availability

The datasets analyzed during the current study are not publicly available due GDP-rules but are available after anonymization from the corresponding author on reasonable request.

## Abbreviations

Alb: Albumin
BAL: Bronchial Lavage
BW: Bronchial Wash
CV: Coefficient of variation
ELISA: Enzyme-linked immunosorbent assay
NaCl: Sodium chloride
ng: nanogram
PEx: Particles Exhaled: i.e. the sample of particles
PExA: Particles in Exhaled Air, i.e. the method
QC: Quality control
SP-A: Surfactant Protein A
wt%: Weight percent
